# Editorial: Novel migraine therapies: consolidating evidence from the real world

**DOI:** 10.3389/fpain.2023.1340268

**Published:** 2023-12-05

**Authors:** Lanfranco Pellesi

**Affiliations:** Clinical Pharmacology, Pharmacy and Environmental Medicine, Department of Public Health, University of Southern Denmark, Odense, Denmark

**Keywords:** CGRP, gepant, headache, lasmiditan, olfactory training, pain

**Editorial on the Research Topic** Novel migraine therapies: consolidating evidence from the real world

Migraine is a debilitating neurological disorder affecting approximately 15% of the global population, making it one of the most prevalent conditions worldwide (1). Symptoms are recurring and include throbbing or pulsating unilateral head pain often accompanied by nausea, vomiting, and sensitivity to light. The burden extends beyond the individual suffering as it has significant social, economic, and healthcare implications. Individuals with no more than 14 headache days per month are characterized by episodic migraine, while the chronic form evolves as a result of increased attack frequency (Mungoven et al.). Chronic migraine is complicated by an excessive or too frequent use of analgesics, leading to a secondary disorder named medication overuse headache (MOH). Overuse of symptomatic medications can produce the paradoxical effect of deteriorating the underlying migraine without a clear explanation (Kebede et al.). Recent advancements in the understanding of migraine have led to the development of therapies that target the calcitonin gene-related peptide (CGRP), a sensory peptide implicated in the initiation and maintenance of migraine episodes (2). Erenumab, galcanezumab, fremanezumab and eptinezumab are monoclonal antibodies (mAbs) that prevent migraine by inhibiting the CGRP pathway. Atogepant, rimegepant, ubrogepant and zavegepant are small-molecule antagonists of the CGRP receptor (or gepants) that are effective in the acute and prophylactic treatment of migraine. Lasmiditan is a new serotonin 5-HT_1F_ receptor agonist that stops acute migraine by blocking CGRP release from trigeminal nerve terminals (3). People who suffer from migraine and MOH are already benefiting from these therapies.

The relevance of real-world investigations in migraine has never been higher. While phase II and phase III clinical trials provide valuable insights into the efficacy and safety of novel therapies, they have strict inclusion and exclusion criteria that do not reflect the true patient population. Real-world studies, however, offer the opportunity to evaluate the effectiveness and tolerability of treatments in a more diverse and representative patient population. Real-world research can offer insightful perspectives on the long-term efficacy of anti-CGRP mAbs, gepants and lasmiditan in lowering the frequency, severity, and duration of migraine episodes. In addition, treatment response predictors affecting treatment adherence and discontinuation may be identified. Certain demographic or clinical features can support the identification of subgroups of individuals who may benefit the most from these therapies. An important factor preventing widespread utilization of anti-CGRP mAbs, gepants and lasmiditan is their high cost (4). For example, regulations make it difficult to treat migraine patients with anti-CGRP mAbs, affecting their ability to adhere to prescribed treatment plans. Several national drug agencies and private insurance programs reimburse anti-CGRP mAbs for a maximum of 12 months in patients with difficult-to-treat migraine. After 1 year of treatment, a period of discontinuation is usually required to determine the necessity of treatment re-initiation (5, 6). In some countries, such as France, there is no reimbursement, and patients must pay anti-CGRP mAbs out of pocket. Assessing the cost-effectiveness of anti-CGRP mAbs, gepants and lasmiditan in the real world is crucial for healthcare decision-makers to allocate resources appropriately and ensure equitable access to these therapies. The evaluation of two therapies combined together with potential synergistic effects in preventing migraine and associated disability is equally relevant (Pellesi). One of the main consequences of high costs is that individuals with migraine seek other strategies, including non-pharmacological therapies (Rundblad et al.). Non-pharmacological approaches can be easily investigated and monitored for efficacy and safety in a real-world environment, including olfactory training, cognitive behavioral therapy, mindfulness meditation, and other relaxation techniques (Gossrau et al.). They are particularly important in children and adolescents because evidence-based treatments are limited in such population. High-quality studies in children and adolescents are characterized by high placebo rate and a significant number of side effects associated with active pharmacological (7, 8). Further real-world research may support the identification of well-tolerated and inexpensive non-pharmacological options that reduce the disability of individuals with migraine.

Real world-studies will provide valuable insights into the effectiveness, long-term outcomes, and predictors of treatment response of new targeted therapies for migraine. They will help identify subgroups of patients who may benefit the most from these therapies and contribute to the development of personalized treatment approaches. However, it is important to acknowledge that real-world studies present certain limitations. The lack of randomization and blinding, inherent in observational studies, may introduce biases and confounding factors that could influence the results. Additionally, data collection in real-world studies relies on electronic health records, patient-reported outcomes, and other sources, which may introduce errors and inconsistencies. Therefore, it is essential to interpret the real-world findings with caution and considering them in conjunction with evidence from randomized controlled trials and other study designs. Future research should continue to emphasize the importance of real-world studies in advancing our understanding of migraine and its consequences, allowing healthcare providers to optimize therapeutic outcomes for individual patients.
